# Ultrasonographic characteristics of thyroid nodule rupture after microwave ablation: Three case reports

**DOI:** 10.1097/MD.0000000000025070

**Published:** 2021-03-05

**Authors:** Peng Tian, Wenyan Du, Xiaoxi Liu, Yiwen Ding, Zekai Zhang, Jing Li, Yanzhen Wang

**Affiliations:** a Department of Ultrasonic; b Department of Science and Education; c Department of Pathology, Zibo Central Hospital, Zibo, Shandong Province, China.

**Keywords:** benign thyroid nodule, microwave ablation, nodule rupture, ultrasonography

## Abstract

**Rationale:**

Thyroid nodule rupture is a rare complication after microwave ablation (MWA). The ultrasonographic characteristics, clinical course, treatment, or prognosis of thyroid nodule rupture after ablation have not been systematically summarized. Three cases with thyroid nodule rupture after MWA were reported in this study, including the characteristic ultrasound images before ablation and after rupture. Meanwhile, we investigated the etiology, diagnosis, treatment and prevention of the rupture. These findings can provide references for the future clinical practice.

**Patients concerns:**

All 3 patients were pathologically diagnosed as benign thyroid nodules by core needle biopsy and then received 1 session of MWA.

**Diagnoses:**

Fourteen days to 1 month after MWA later, all 3 patients presented with abrupt neck pain and swelling, and 1 of them had a fever. Ultrasound examinations shared common features that the rupture of thyroid capsule and a soft-tissue mass with unclear margin in front of the thyroid gland, which connected with the post-ablation nodule. Three patients were diagnosed as thyroid nodule ruptures.

**Interventions:**

All 3 patients received conservative management after the ruptures. With the treatment of intravenous antibiotics for 1 week, the neck swelling of patients 1 and 2 both disappeared. The aggravation of neck swelling was found in patient 3. Ultrasonography of the neck revealed irregular fluid echo in the soft-tissue mass, suggesting abscess formation. Aspiration and irrigation were performed. The neck swelling regressed gradually over another 2 weeks with the treatment of antibiotics. Two months after ablation, ultrasound examination showed that the mass had completely disappeared.

**Outcomes:**

None of the 3 patients underwent open surgery due to thyroid nodule rupture. At 1-year follow-up, the volume reduction rate of thyroid nodules in 3 patients were as follows: 100%, 98.1% and 90.7%.

**Lessons:**

Nodule rupture is a rare but severe complication after MWA of the thyroid nodules. The diagnosis can be confirmed by clinical symptoms and ultrasound examination, and most nodule ruptures could be cured with conservative treatment. Grasping the characteristics of ultrasound imaging during the course of disease, and dynamically assessing course of disease progression by ultrasonography could avoid unnecessary imaging examinations or invasive procedures.

## 1 Introduction

Thyroid nodules are a common disease in endocrine system.^[1]^ More than 95% of thyroid nodules are benign, most of which do not need any treatment; however, clinical intervention may be required when symptoms of neck compression or aesthetic effects were indicated.^[2]^ With the development of minimally invasive techniques, thermal ablation therapies including radiofrequency ablation, laser ablation and microwave ablation (MWA) significantly reduce the volume of thyroid nodules or even disappear entirely.^[3]^ This emerging method has multiple effects of beauty, elimination of compression symptoms, avoidance of surgery, and reaching a global consensus.^[4,5]^ Thermal ablation of benign thyroid nodules (BTNs) has many advantages, such as minimally invasive, no surgical incision, fewer complications, and widely recognized by clinicians and patients. At present, although the incidence of complications has been reduced utilizing moving-shot technique or liquid-isolating maneuver, severe complications may still occur, including recurrent laryngeal nerve injury, nodule rupture, or massive hemorrhage.^[6]^ So far, the previous reports on ultrasonographic characteristics, clinical course, treatment, or prognosis of thyroid nodule rupture after ablation were limited. This retrospective study revealed the ultrasonographic characteristics, clinical symptoms, treatment, and outcome of 3 thyroid nodule rupture cases after MWA and inferred the causes and mechanisms of thyroid nodule rupture. These findings could provide clinical experience for the prevention, diagnosis and treatment of thyroid nodule rupture after ablation.

## 2 Case report

A total of 1092 patients with BTNs were treated with ultrasonic guided MWA between May 2014 and December 2019 in our department. All ablation procedures were performed by 1 specialized radiologist with 10 years of experience. All nodules reported were pathologically diagnosed as BTNs by fine needle aspiration cytology or core needle biopsy before MWA. All ablations were performed using an ECO-100C microwave generator (ECO Medical Equipment Co Ltd, Jiangsu, China) and a 100-mm-length, 18G, 3-mm active-tip internally cooled ablation needle, at 2450 MHz, with power of 30 W. Three cases received 1 session of ablation in our report had no history of surgical treatment and no underlying diseases such as diabetes and hypertension. This report was approved by the Ethics Committee of Zibo Central Hospital and informed written consents from the 3 patients.

The nodule volume was calculated by measuring the 3 largest perpendicular diameters (a, b, c), with b and c perpendicular to each other, V = π× abc /6.

Volume Reduction Rate  = [(Volume before ablation - Volume during follow-up)/ Volume before ablation] × 100%;

### 2.1 Case 1

**Figure 1:**
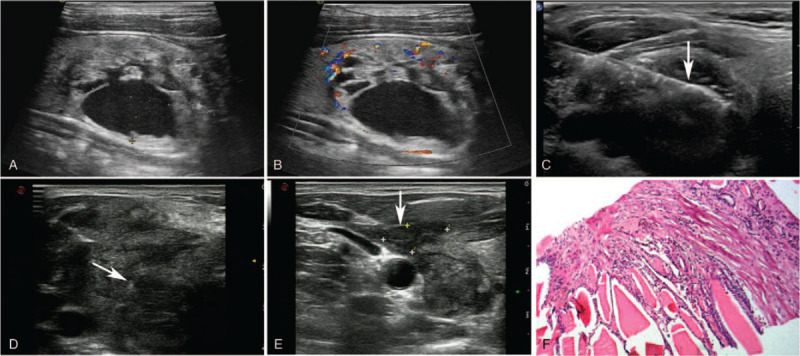
Case 1. (A) The size of BTN in the right thyroid gland was about 41.9 × 35.2 × 26.1 mm. (B) CDFI showed abundant blood flow signals around the nodule and in the solid portion. (C) The microwave ablation needle (arrow) transmitted in the thyroid nodule. (D) The continuity of the anterior capsule of the right thyroid gland was interrupted (arrow), and the size of the cervical soft-tissue mass was about 32.1 × 28.6 × 22.8 mm. (E) After conservative treatment for 1 week, ultrasonography showed that the inferior echogenic area (arrow) in the front of the right thyroid was about 14.4 × 12.1 × 6.8 mm. (F) The pathological report of thyroid nodule by CNB, Hematoxylin-eosin (HE) staining (magnitude: 20 × 10).

A 41-year-old man was admitted to hospital due to a lump in his neck. Ultrasound examination indicated a single, clear margin nodule with predominantly solid components (>50% solid portion, 41.9 × 35.2 × 26.1 mm) in the anterior portion of the right thyroid gland (Fig. 1). Color Doppler Flow Imaging (CDFI) showed abundant blood flow signals around the nodule and in the solid portion. On December 8, 2016, he initially underwent microwave ablation of thyroid nodule with a power of 30W for 387 s of active time (AT). He was admitted to hospital again due to cervical swelling 14 days after MWA. Ultrasound examination showed a partial capsule in the anterior thyroid and a soft-tissue mass with unclear margin in the back of the muscle layer on the right side of the neck, about 32.1 × 28.6 × 22.8 mm in size, which connected with the post-ablation nodule. This patient received the conservative treatment, and the symptom of cervical swelling were obviously relieved 7 days later.

### 2.2 Case 2

**Figure 2:**
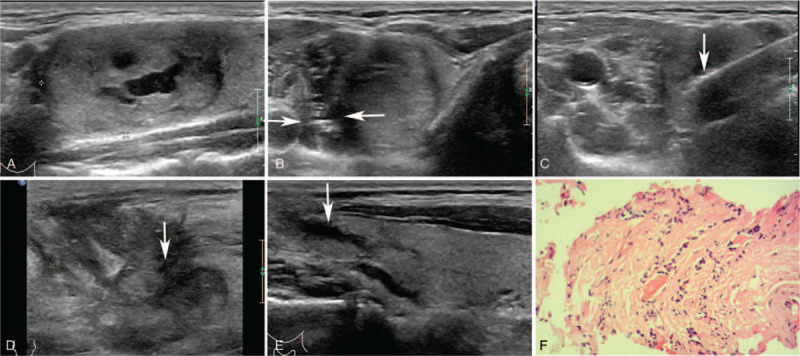
Case 2. (A) The size of BTN in the right thyroid gland was about33.8 × 27.9 × 20.3 mm. (B) Fluid isolation zone formed by injection of normal saline (between two arrows). (C) The microwave ablation needle (arrow) transmitted in the thyroid nodule. (D) The continuity of the anterior capsule of the right lobe of the thyroid gland was interrupted (arrow), and the size of the cervical soft-tissue mass was about 42.3 × 35.2 × 28.7 mm. (E). Six months after ablation, ultrasonography revealed an elongated low echogenicity in the right lobe of the thyroid (arrow). (F) The pathological report of thyroid nodule by CNB, Hematoxylin-eosin (HE) staining (magnitude: 20 × 10).

A 43-year-old man had a single, clear margin, predominantly solid nodule (>80% solid portion, 33.8 × 27.9 × 20.3 mm) in the superior portion of the right lateral thyroid lobe through physical examination (Fig. 2). CDFI showed circular blood flow signals around the nodule and poor signals inside. On September 12, 2017, he was treated with MWA of thyroid nodule with a power of 30W for 332 s of AT. One month after MWA later, his neck developed local swelling and tenderness. Ultrasound examination suggested that a soft-tissue mass at the front of the right thyroid gland, which was approximately 42.3 × 35.2 × 28.7 mm in size. Meanwhile the continuity of the anterior capsule of the right lobe of thyroid gland was found to be interrupted and the post-ablation nodule connected with the anterior soft-tissue mass. With 1-week conservative management the symptoms of cervical swelling and tenderness were gradually lessened.

### 2.3 Case 3

**Figure 3:**
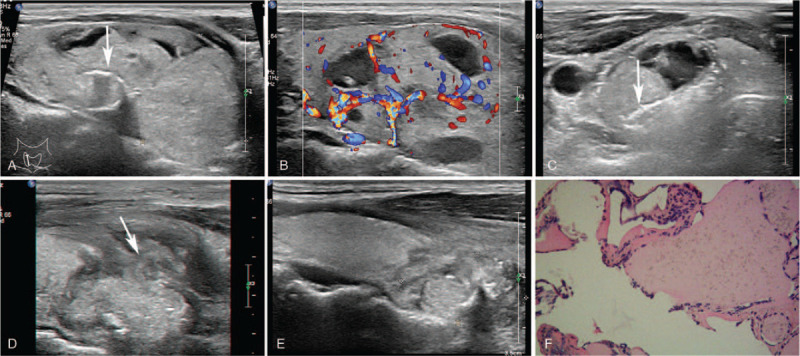
Case 3. (A) The size of the benign tumor in the right lobe of the thyroid was about 55.1 × 40.9 × 27.5 mm, with arc-shaped calcification inside (arrow). (B) CDFI shows circulatory blood flow signals around the nodule and abundant blood flow signals within the nodule. (C) The microwave ablation needle (arrow) transmitted in the thyroid nodule. (D) The continuity of the anterior capsule of the right lobe of the thyroid gland was interrupted (arrow), and the size of the cervical soft-tissue mass was about 40.3 × 25.7 × 28.2 mm. (E) After ablation for six months, ultrasound examination showed the size of the post-ablation nodule was 25.1 × 20.5 × 14.2 mm, and the anterior inferior echoic area of thyroid disappeared. (F) The pathological report of thyroid nodule by CNB, Hematoxylin-eosin (HE) staining (magnitude: 20 × 10).

A 33-year-old female patient presented with progressive enlargement of thyroid nodule for three years. Recently, she was admitted to hospital with symptoms of neck compression. Ultrasonic examination revealed a large solid mass in the right lobe of thyroid, which was approximately 55.1 × 40.9 × 27.5 mm in size, with clear margin, regular morphology, less homogeneous echo, and arc-shaped calcification in the center (Fig. 3). CDFI showed circulatory blood flow signals around the nodule and abundant signals inside. The nodule was pathologically confirmed as a benign tumor, but the patient refused to receive a surgical resection. On November 22, 2019, she was treated with MWA of thyroid nodule with a power of 30W for 826 s of AT. One week after MWA, she felt swelling in her neck and complained of dysphagia and odynophagia. Sonography showed that the thyroid capsule was intact, and the size of the post-ablation nodule was about 46.8 × 41.5 × 29.1 mm, which with clear margin, full shape and mixed internal echoes. The examination showed no blood flow signal inside the nodule and a little blood flow signals around the nodule on CDFI. The cervical swelling was considered to be a normal reaction after MWA clinically, so no clinical treatment was performed on this patient. Two weeks after MWA later, the patient returned with fever, swelling and tenderness of the neck. Sonography demonstrated the breakdown of the anterior thyroid capsule and a poorly demarcated soft-tissue mass in front of the thyroid gland, approximately 40.3 × 25.7 × 28.2 mm in size. The post-ablation nodule became irregular and connected with the anterior soft-tissue mass. The patient's neck continued swelling with increased redness, and a sense of fluctuation was detected, despite receiving 1-week intravenous antibiotics. Sonography of the right neck showed fluid echo in the soft-tissue mass, suggesting the abscess formation. 2 ml semisolid blood clot was aspirated by the puncture needle (16G) for bacterial culture and drug sensitivity test. We found no bacterial growth was observed in the sample. After sustaining antibiotic treatment for 2 weeks, the neck swelling was gradually reduced, and sonography showed the soft-tissue mass of the right neck was regressed, with no obvious fluid echo inside.

## 3 Discussion

MWA for BTNs is a minimally invasive technique that has been used as a safe and effective alternative for the treatment of BTNs.^[7]^ The previous literature reported that the volume reduction rate of BTNs after 12 months of MWA was 92.4%.^[8]^ The incidence of MWA complications is much lower than that of surgical resection. However, there is still a risk of nodule rupture after ablation. Thyroid nodule rupture after ablation is defined as the breakdown of the thyroid capsule and communication between the post-ablation nodule and external thyroid tissue.^[9]^ In our study, foreign literatures of nodule rupture after ablation were retrieved, and summarized in Table 1.^[10–14]^ A total of 27 cases of nodule ruptures after ablation were reported in 5 papers with an incidence of 0.2% to 2.4%. In our study, the incidence of thyroid nodule spontaneous rupture after MWA was 0.27%, which was basically consistent with the incidence in Table 1. It was generally believed that physicians with extensive experience in clinical interventional therapy could reduce the incidence of nodule rupture.

**Table 1:** Clinical manifestations and treatment of thyroid nodule ruptures after ablation reported in previous literature.

No.	First Authors (PubMed Unique Identifier, year)	Number of nodule ruptures	Incidence of nodule ruptures	The mean volume of nodules before MWA (ml)	Time to rupture from MWA (day)	treatment
1	Kim C et al^[10]^ (PMID: 27975148, 2017)	3	0.3%	37.5	50 to 145	abscess drainage (1 case) Conservative treatment (2 cases)
2	Baek JH et al^[11]^ (PMID: 21998044, 2012)	3	0.2%	14.9	20 to 50	Conservative treatment (2 cases) Unilateral lobectomy (1 case)
3	Chung SR et al^[12]^ (PMID: 31884742, 2019)	12	0.3%	17.1	11 to 156	Conservative treatment (8 cases) incision and drainage or aspiration (4 cases)
4	Shin JH et al^[13]^ (PMID: 21920870, 2011)	6	0.4%	22.5	9 to 60	incision and drainage (2 cases) Unilateral lobectomy (1 case) Conservative treatment (3 cases)
5	Valcavi R et al^[14]^ (PMID: 20929405, 2010)	3	2.4%	21.4	14 to 21	Conservative treatment (3 cases)

The clinical information of 3 patients in our study is shown in Table 2. The preoperative imaging features of thyroid nodules in all 3 patients were single and large, with a mean volume of 20.8 ml. The characteristics included that the nodules involved the thyroid sub-capsule, and there was no normal thyroid tissue between the thyroid nodule and the anterior cervical tissue, and the anterior capsule was convex. The composition of thyroid nodule can be solid or solid-cystic. CDFI generally showed abundant blood flow signals around the nodule and inside the solid portion. J.H. Shin et al.^[13]^ reported that the average volume of the preoperative nodules was greater than 17. 1 ml in the 6 cases of nodule ruptures after ablation, and the preoperative ultrasound image features were basically consistent with those of the cases in our study. Therefore, we believe that the relatively large volume, adjacent to the thyroid capsule, and abundant blood flow of thyroid nodules are high risk factors for nodule rupture after ablation.

**Table 2:** Clinical data, ultrasound image, and treatment of the 3 patients with nodule rupture after MWA.

Patient no.	Sex	Age	Volume	Nodule number	SonographicComposition	Power(W)/AT(s)	Time to rupture from MWA (d)	Treatment	Outcome
1	M	41	20.1ml	1	Predominantly solid	30w/387s	21	intravenous antibiotics	Complete recovery
2	M	43	10.1ml	1	Predominantly solid	30w/332s	30	intravenous antibiotics	Complete recovery
3	F	33	32.4ml	1	solid	30w/826s	14	intravenous antibiotics and aspiration	Complete recovery

In our study, the clinical manifestations of 3 patients with thyroid nodule rupture after MWA were sudden neck swelling and tenderness, and only one patient with fever, which was also consistent with the clinical manifestations of the cases reported in Table 1. The ultrasonographic characteristics include unclear marginal soft-tissue mass around the thyroid gland, the partial thyroid capsule and communication between the post-ablation nodule and external thyroid tissue. Computed tomography (CT) scan image shows thyroid capsule rupture and local cellulitis of cervical soft tissue.^[15]^ The clinician could make a correct diagnosis based on clinical symptoms and ultrasound or CT examination. However, it should be distinguished from the infectious mass in cervical soft tissue. The infectious mass generally shows a mixed echo in soft tissue in front of thyroid, with unclear boundary, heterogeneous echogenicity and with fluid sonolucent area or not on sonography. CDFI showed that blood flow signals are abundant. The main differentiating point is that the thyroid capsule is intact in patients with soft-tissue infective mass. The nodule rupture in our study occurred 14 to 30 days after MWA, and it has been reported in the literature that it usually occurred 7 to 90 days, or even 156 days after ablation.^[16]^ Based on the rupture localization, thyroid nodule rupture is classified into 3 types: anterior, posterolateral and medial types.^[17]^ The anterior type is the most common, followed by posterolateral and then medial type. The rupture site may be related to the location of the thyroid nodules. In our study, the thyroid nodule ruptures of 3 patients were all anterior type, thyroid nodules of whom were all located under the anterior capsule. For the patients with posterolateral and medial rupture, mediastinal hematoma is easy to be misdiagnosed by ultrasound examination only, which needs to be scanned by CT.^[18]^

The mechanism of thyroid nodule rupture after ablation is still unclear, following with 2 views. First, delayed hemorrhage occurs in the post-ablation nodules, which may be caused by micro-vessels leakage at the edge of the nodules. That can increase the inner pressure the nodule and the volume expansion, inducing laceration of the weak point in the thyroid capsule, and the nodule rupture finally.^[19]^ Second, sudden severe cough, sneeze, hiccups, forced pressure and other external factors increase the inner pressure of the nodule, leading to the post-ablation nodule rupture.^[15]^ Although there is no evidence of leakage in the ablation syringe, the thyroid capsule at the ablation syringe is prone to rupture after ablation. The thyroid capsule has been damaged, especially when the needle ablation was performed to reduce bleeding. We believe that a careful preoperative evaluation should be done to prevent nodule ruptures after ablation. Compared to other thyroid nodules, the nodules with larger size, adjacent to the thyroid capsule, and abundant blood flow distribution have a higher risk of post-ablation rupture. Occlusion of the nodule's circumferential ring by ablation can reduce the risk of intraoperative and/or delayed postoperative bleeding. Because of the importance of post-ablation care, patients are required to rest, avoid neck massage, fatal pressure, severe cough and other behaviors that may cause nodule rupture. Patients with ruptured nodules should be advised to protect the neck to avoid further compression resulting in more severe results. Patient 3 in this study presented neck swelling and discomfort 1 week after ablation. The post-ablation nodule was plump in shape and did not rupture on sonography yet. But sonography second-week after MVA indicated the spontaneous rupture of the nodule. Therefore, we supposed that the occurrence of neck swelling after ablation and the full-form nodules on ultrasound imaging, may be the special appearance before the nodule ruptures. For those patients with the underlying risk of nodule rupture, subsequent examinations with a higher frequency should be performed to prevent the rupture.

In our study, all 3 patients received the conservative treatment. The symptoms of patient 1 and 2 were relieved a week later, whereas patient 3 experienced aggravated abscess formation. No bacterial infection was detected with the bacterial culture test of the pus subsequently. After 2 weeks of continuous antibiotic treatment, the symptoms gradually disappeared. The inflammatory reaction and abscess formation in the neck may be due to a large quantity of apoptotic cells aggregating in the ablation lesion, necrosis liquefaction, and the infiltration of endotoxin into the surrounding interstitial tissue. In Table 2, 27 cases of ruptured nodules were reported, among which 18 cases received conservative treatment (66.67%), 7 cases received aspiration and drainage (25.92%), and 2 cases received surgery (7.41%). The bacterial culture results of 7 cases with drainage showed no bacterial infection, which was consistent with the result of our study. Ultrasonographic characteristics, patient symptoms, and recovery time are related to initial volume of nodules, rupture size, and immune response.^[20]^ The larger nodule and more severe rupture could lead to the more obvious the symptoms and longer clinical course. Because hemorrhage in most nodule ruptures can be self-limiting and aseptic, we recommend conservative treatment for these patients. With 2 weeks of conservative treatment, the neck swelling symptoms usually alleviate and the cervical soft-tissue mass gradually regressed. During the conservative treatment, the patient's symptoms and changes in ultrasound images were closely observed. If the patient had increased neck swelling and fever, and the range of neck mass in ultrasound images was increased with fluid echo, puncture drainage or surgical treatment is ought to be actively performed.

## 4 Conclusion

Nodule rupture is a rare but severe complication after MWA of thyroid nodule. Large-sized, adjacent to thyroid capsule, and abundant blood flow signal in nodules are the risk factors for post-ablation rupture. Thyroid nodule rupture occurs between 7 and 156 days after ablation. The possibility of thyroid nodule rupture should be considered if there are sudden neck swelling and tenderness after MWA. The Ultrasonographic characteristics including the partial thyroid capsule and cervical soft-tissue mass connecting with thyroid post-ablation nodule, can be used to confirm the diagnosis of thyroid nodule rupture. Conservative treatment is recommended for nodule ruptures after ablation, meanwhile the changes of ultrasonographic image characteristics are dynamically detected by ultrasound examination. The swelling of neck in most patients regresses and gradually improves after approximately 2 weeks. If conservative treatment fails, puncture drainage or surgery should be performed promptly. Mastering the risk factors of nodule rupture, clinical symptoms, characteristics of disease progression and ultrasonographic features while improving the microwave ablation technology of thyroid nodules can reduce the incidence of nodule rupture after ablation.

## Author contributions

**Data curation:** Peng Tian, Wenyan Du, Jing Li, Yanzhen Wang.

**Formal analysis:** Peng Tian.

**Funding acquisition:** Peng Tian.

**Investigation:** Wenyan Du, Yiwen Ding, Zekai Zhang.

**Methodology:** Wenyan Du, Xiaoxi Liu, Yiwen Ding, Zekai Zhang.

**Project administration:** Xiaoxi Liu, Yiwen Ding, Yanzhen Wang.

**Resources:** Wenyan Du, Xiaoxi Liu, Yiwen Ding, Zekai Zhang, Jing Li, Yanzhen Wang.

**Software:** Zekai Zhang.

**Supervision:** Xiaoxi Liu.

**Validation:** Yanzhen Wang.

**Writing – original draft:** Peng Tian, Jing Li, Yanzhen Wang.

**Writing – review and editing:** Peng Tian.
